# Supplementary material for: Primary care pharyngotonsillitis complications following absent or deferred antibiotic treatment across the COVID 19 pandemic
Source: Commun Med (Lond). 2026 Apr 1;6:185. doi: 10.1038/s43856-026-01564-z (PMC13046787; doi:10.1038/s43856-026-01564-z)

Supplementary information for

Primary Care Pharyngotonsillitis Complications Following Absent or Deferred Antibiotic Treatment Across the COVID 19 Pandemic

Ailiana Santosa, Julius Collin, Elin Dahlén, Anders Lignell, Maria Furberg, Anders Ternhag, Rickard Ljung, Fredrik Nyberg

Suppl Table 1. Codes used for identification of pharyngotonsillitis and infectious complications observed within 30 days of the pharyngotonsillitis diagnosis.

Diagnosis[∞]	ICD-10 codes		Complication[§]	ICD-10 codes
Pharyngotonsillitis	J02, J02.0, J02.8, J02.9, J03, J03.0, J03.8, J03.9, J01AA02, J01CE02, J01CA04, J01CR02, J01DB, J01DC, J01DD, J01DE, J01FA, J01FF01	→	Peritonsillar abscess	J36, J36.9
		→	Retro-parapharyngeal abscess	J390
		→	Necrotizing fasciitis	M726
		→	Streptococcal sepsis	A400<br>A403<br>A409
		→	Rheumatic fever	I00, I00.9<br>I01, I01.9

Footnote: [∞] pharyngotonsillitis diagnosis retrieved from primary healthcare registers in Stockholm and Västra Götaland regions between 1 January 2018 and 31 December 2023.
[§] complications occurred within 30 days after pharyngotonsillitis diagnosis

Suppl Table 2. ATC codes used to classify antibiotics commonly prescribed for the treatment of pharyngotonsillitis

Respiratory tract antibiotics	ATC codes	Proportion (%)
Penicillin V		
Phenoximethylpenicillin (PcV)	J01CE02	89.5%
Other		
Doxycycline	J01AA02	0.96%
Amoxicillin	J01CA04	1.06%
Amoxicillin/Clavulanic acid	J01CR02	0.26%
Cephalosporines	J01DB-DE	0.82%
Macrolides	J01FA01, J01FA09, J01FA10	1.55%
Clindamycin	J01FF01	5.85%

Suppl Table 3. ICD-codes used for Charlson Comorbidity Index.

Comorbidity	ICD-10 codes
Myocardial infarction	I21, 122, 125.2
Congestive heart failure	I11.0, I13.0, I13.2, I25.5, I42.0, I42.6, I42.7, I42.8, I42.9, I43, I50,
Peripheral vascular disease	I70, I71, I73.1, I73.8, I73.9, I77.1, I79.0, I79.2, K55
Cerebrovascular disease	G45, I60, I61, I62, I63, I64, I67, I69,
COPD	J43, J44,
Other chronic pulmonary disease	J41, J42, J45, J46, J47, J60, J61, J62, J63, J64, J65, J66, J67, J68, J69, J70
Rheumatic disease	M05; M06, M12.3, M07.0–3, M08, M13, M30, M31.3-M31.6, M32, M33, M34, M35.0, M35.1, M35.3, M45-46
Dementia	F00, F01, F02, F03, F05.1, G30, G31.1, G31.9
Hemiplegia, tetraplegia	G11.4, G80, G81, G82, G83.0-G83.3, G83.8
Diabetes	E10.0, E10.1, E11.0-E11.1, E12.0-E12.1, E13.0-E13.1, E14.0-E14.1
Diabetes with end organ damage	E10.2, E10.3, E10.4, E10.5, E10.7, E11.2-E11.7, E12.2-E12.7, E13.2-E13.7, E14.2-E14.7,
Moderate or severe kidney disease	N03.2-N03.7, N05.2-N05.7, N11, N18, N19, N25.0, I12.0, I13.1, Q61.1-Q61.4, Z49, Z94.0, Z99.2
Mild liver disease	B15-B19, K70.3, K73, K74.6, K70.3, K75.4
Moderate or severe liver disease	R18, I85.0, I85.9, I98.2, I98.3
Peptic ulcer disease	K25, K26, K27, K28
Any malignancy including leukemia and lymphoma	C00-C97 not C87, C00, C01, C02, C03, C04, C05, C06, C07, C08, C09, C10, C11, C12, C13, C14, C15, C16, C17, C18, C19, C20, C21, C22, C23, C24, C25, C26, C30, C31, C32, C33, C34, C37, C38, C39, C40, C41, C43, C45, C46, C47, C48, C49, C50, C51, C52, C53, C54, C55, C56, C57, C58, C60, C61, C62, C63, C64, C65, C66, C67, C68, C69, C70, C71, C72, C73, C74, C75, C76, C81, C82, C83, C84, C85, C86, C88, C90, C91, C92, C93, C94, C95, C96, C97
Metastatic cancer	C77, C78, C79, C80
Hiv/Aids	B20, B21, B22, B23, B24, F02.4, O98.7, R75, Z11.4, Z21.9, Z71.1

Suppl Table 4. Definitions for immunosuppression (i.e. autoimmune disease or immunosuppressive treatment)

Autoimmun disease / immunosuppression:	ICD-10 or ATC codes
Autoimmune disease (ICD-10 codes)	D510, D590, D591, D690, D693, D86, E035, E039, E050, E055, E059, E063, E065, E271, E272, E310 G04, G131, G35, G36, G61, G700 H20 I00-I02 K50, K51, K732, K743, K900 L10, L12, L130, L40, L63, L80 M05-06, M08, M30, M311, M313, M315-7, M32-34, M350-M353, M358-M359, M45, M60<br>C00-96 (NOT C44), D70-72, D730, D81-84<br>E10-14
Immunosuppressive treatment (ATC codes)	L04AA selective immunosuppressants<br>L04AB TNF-alfa inhibitors<br>L04AC Interleukin inhibitors<br>L04AD Calcineurin inhibitors<br>L04AX Other immunosuppressants<br>L01BA01 Methotrexate<br>L01AA01 Cyclophosphamide<br>H02AB Glucocorticoids

Suppl Table 5. Characteristics of sociodemographics, prior comorbidities, healthcare utilization, COVID-19 vaccination and drug prescriptions among pharyngotonsillitis between 1 January 2018 and 31 December 2023 and received antibiotic treatment, by pandemic period

Characteristics	Pre-pandemic	Pandemic	Post-pandemic
	n=58,109	n=40,369	n=43,473
Sex, male	22,477 (38.7%)	15,107 (37.4%)	16,279 (37.4%)
Age group (years)			
12-24	19,594 (33.7%)	11,771 (29.2%)	10,607 (24.4%)
25-39	19,506 (33.6%)	14,275 (35.4%)	17,411 (40.1%)
40-69	16,581 (28.5%)	12,473 (30.9%)	13,484 (31.0%)
70+	2,428 (4.2%)	1,850 (4.6%)	1,971 (4.5%)
Education level			
Primary school	14,905 (25.7%)	10,820 (26.8%)	11,268 (25.9%)
Secondary school	6,500 (11.2%)	4,664 (11.6%)	4,610 (10.6%)
Tertiary school<3 years	15,537 (26.7%)	10,149 (25.1%)	11,332 (26.1%)
Tertiary school≥ 3years	21,167 (36.4%)	14,736 (36.5%)	16,263 (37.4%)
Marital status			
Not married	41,119 (70.8%)	29,539 (73.2%)	31,183 (71.7%)
Married/register partner	16,990 (29.2%)	10,830 (26.8%)	12,290 (28.3%)
Country of birth			
Nordic countries	46,681 (80.3%)	32,045 (79.4%)	34,672 (79.8%)
Other countries	11,428 (19.7%)	8,324 (20.6%)	8,801 (20.2%)
Vaccination			
None	8,143 (14.0%)	5,324 (13.2%)	6,664 (15.3%)
1 vaccine	1,549 (2.7%)	1,159 (2.9%)	1,204 (2.8%)
2 vaccine	17,273 (29.7%)	12,424 (30.8%)	12,871 (29.6%)
Booster	31,144 (53.6%)	21,462 (53.2%)	22,734 (52.3%)
Number of primary care visits			
One visit	44,981 (77.4%)	29,412 (72.9%)	32,790 (75.4%)
Two visits	10,297 (17.7%)	8,290 (20.5%)	8,231 (18.9%)
Three visits	2,274 (3.9%)	2,094 (5.2%)	1,950 (4.5%)
Four or more visits	557 (1.0%)	573 (1.4%)	502 (1.2%)
Charlson comorbidity index			
0	46,335 (79.7%)	31,314 (77.6%)	34,658 (79.7%)
1	5,252 (9.0%)	3,790 (9.4%)	4,019 (9.2%)
2-3	1,924 (3.3%)	1,448 (3.6%)	1,603 (3.7%)
4-5	171 (0.3%)	187 (0.5%)	194 (0.4%)
≥6	4,427 (7.6%)	3,630 (9.0%)	2,999 (6.9%)
Autoimmune/immunosuppression	4,168 (7.2%)	3,572 (8.8%)	4,450 (10.2%)
Immunosuppressive treatments			
Selective immunosuppressants	45 (0.1%)	71 (0.2%)	93 (0.2%)
TNF-alfa inhibitors	338 (0.6%)	292 (0.7%)	479 (1.1%)
Interleukin inhibitors	90 (0.2%)	78 (0.2%)	129 (0.3%)
Other immunosuppressants	497 (0.9%)	488 (1.2%)	672 (1.5%)
Glucocorticoids	3,410 (5.9%)	4,326 (10.7%)	5,998 (13.8%)
Cytostatic	96 (0.2%)	97 (0.2%)	212 (0.5%)

Footnote: Pre-pandemic: 1 Jan 2018-31 Jan 2020; Pandemic: 1 Feb 2020-28 Feb 2022; Post pandemic: 1 Mar 2022-31 Jan 2024

Suppl Table 6. Proportion of complications observed within 30 days among pharyngotonsillitis patients aged 12 years and above between1 January 2018 and 31 January 2024, stratified by pandemic period.

Period	Antibiotic treatment	Complications	Type of complications				
			Peritonsillar abscess	Retropharyngeal and parapharyngeal abscess	Rheumatic fever	Necroti-zing fasciitis	Bacteraemia
Pre-pandemic	Yes	1124 (1.38%)	1100 (1.35%)	29 (0.04%)	0	<5(0)	10 (0.01%)
	No	222 (0.38%)	208 (0.38%)	17 (0.03%)	0	<5(0)	<5(0)
Pandemic	Yes	692 (2.58%)	683 (2.55%)	12 (0.04%)	0	0	<5(0)
	No	169 (0.42%)	154 (0.38%)	14 (0.03%)	0	0	<5(0)
Post-pandemic	Yes	879 (1.92%)	864 (1.89%)	21 (0.05%)	0	<5(0)	<5(0)
	No	221 (0.51%)	215 (0.49%)	7 (0.02%)	0	<5(0)	<5(0)

Footnote: Pre-pandemic: 1 Jan 2018-31 Jan 2020; Pandemic: 1 Feb 2020-28 Feb 2022; Post pandemic: 1 Mar 2022-31 Jan 2024

Suppl Table 7. Sensitivity analysis: Crude and adjusted odds ratios for the association between antibiotic dispensing within 5 days (no vs. yes) and antibiotic type (other antibiotics vs. penicillin V) with complications within 30 days among patients diagnosed with pharyngotonsillitis in primary healthcare in Stockholm and Västra Götaland Regions, Sweden, overall and by pandemic period.

Period	Antibiotic treatment (No vs Yes)		Type of antibiotic[a] (Other vs Penicillin)	
	Crude OR(95%CI)	aOR[b] (95%CI)	Crude OR(95%CI)	aOR[b] (95%CI)
Overall	0.11 (0.10-0.12)	0.11 (0.09-0.12)	1.85 (1.69-2.03)	1.95 (1.78-2.15)
Pre-pandemic	0.12 (0.10-0.15)	0.12 (0.10-0.15)	2.13 (1.86-2.45)	2.18 (1.90-2.51)
Pandemic	0.07 (0.05-0.08)	0.07 (0.06-0.09)	1.73 (1.44-2.09)	1.82 (1.51-2.20)
Post pandemic	0.12 (0.10-0.14)	0.13 (0.10-0.15)	1.60 (1.35-1.91)	1.74 (1.45-2.08)

Footnote: aOR: adjusted odds ratio; CI: confidence interval

[a] analysis only including those who received antibiotic treatment

[b] adjusted for age, sex, sociodemographics, Charlson comorbidity index, vaccination status, primary care visits, and immunosuppressive treatments

Supp.Table 8. Sensitivity analysis: Association between antibiotic treatment (No vs Yes) and type of antibiotic (other antibiotics vs penicillin) with specific complications within 30 days among pharyngotonsillitis patients in Stockholm and Västra Götaland regions in Sweden.

Type of complications	Antibiotic treatment (No vs Yes)		Type of antibiotic[a] (Other vs Penicillin)	
	Crude OR, 95% CI	aOR[b], 95% CI	Crude OR, 95% CI	aOR[b], 95% CI
Peritonsillar abscess	0.10 (0.09-0.11)	0.10 (0.09-0.11)	1.81 (1.65-1.99)	1.91(1.73-2.11)
Retropharyngeal & parapharyngeal abscess	0.30 (0.19-0.49)	0.32 (0.20-0.52)	6.57(4.21-10.2)	6.57(4.18-10.3)
Rheumatic fever	-	-	-	-
Necrotizing fasciitis	1.41 (0.28-7.10)	0.85(0.17-4.30)	-	-
Bacteremia	0.37 (0.15-0.89)	0.60 (0.26-1.35)	0.97 (0.22-4.20)	0.81 (0.18-3.62)

Footnote: aOR: adjusted odds ratio; CI: confidence interval

[a] analysis only including those who received antibiotic treatment

[b] adjusted for age, sex, sociodemographics, Charlson comorbidity index, vaccination status, primary care visits, and immunosuppressive treatments

Supp. Figure 1. Monthly trends in antibiotic prescriptions (all ATC types) among patients diagnosed with pharyngotonsillitis in Stockholm and Västra Götaland Regions, Sweden, 2018–2024.

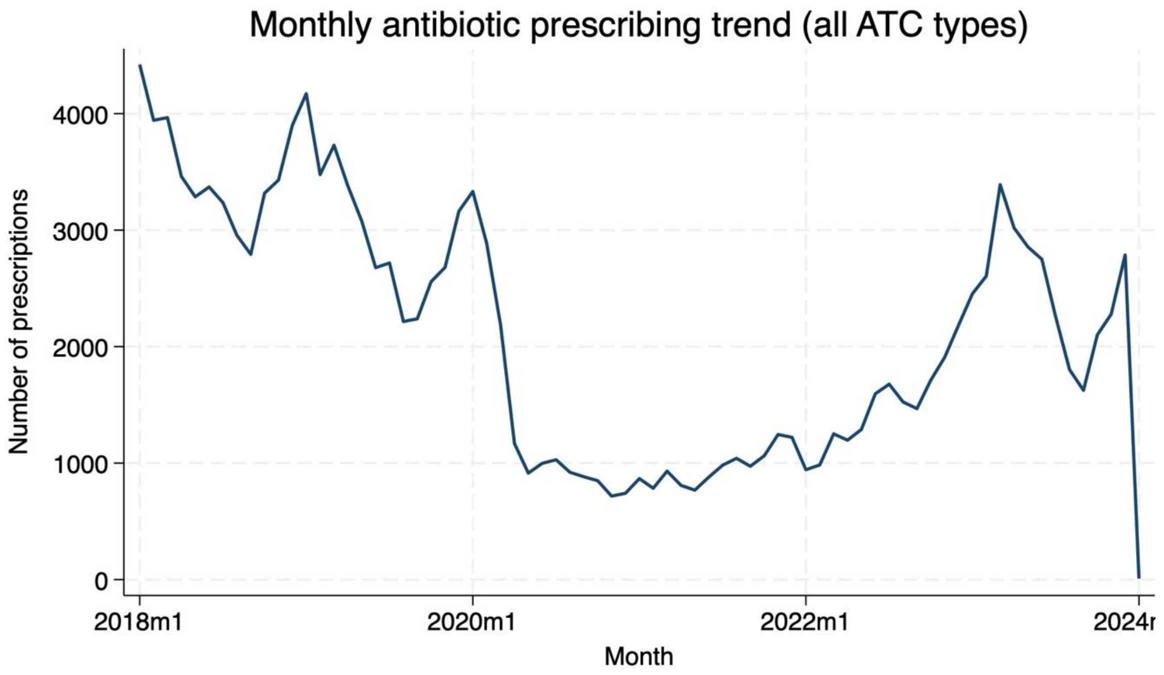

Supplement: Supplementary file 2 — Supplementary Information [file 43856_2026_1564_MOESM2_ESM.pdf]
